# Testosterone Therapy for Women: Still Many Questions to be Answered

**DOI:** 10.1055/s-0042-1742619

**Published:** 2022-01-29

**Authors:** Cristina Laguna Benetti-Pinto

**Affiliations:** 1 Departamento de Tocoginecologia, Faculdade de Ciências Médicas, Universidade Estadual de Campinas, Campinas, SP, Brazil

In recent years, the biological role of androgens in the female organism has aroused increasing interest. Women in the reproductive period and after menopause produce androgens that contribute to ovarian function, sexual function, bone metabolism, cognition, among other actions. Despite the evidence of involvement of its excess in reproductive health disorders, studies on its deficiency have increased.

Androgen levels in the female body vary with age. In addition to peripheral conversion mechanisms, ovaries and adrenals are responsible for the production and maintenance of circulating androgenic levels. After a peak at between 18 and 24 years of age, a slow decline begins during the reproductive period, around 30 years of age, and at 70–80 years of age, serum levels of androgens will represent 10 to 20% of levels found at the age of 30. In this process, menopause by itself does not represent a moment of accentuated androgenic decline. 1 2

Testosterone is the most potent androgen circulating in the female body and generally the most dosed. Prescription rates have increased recently under the justification of alleviating the symptoms arising from its lack. The question is: how to characterize androgen deficiency in women? What is the evidence for its use?

Symptoms of androgen deficiency at different stages of life are nonspecific. Complaints of reduced arousal, fantasies, sexual desire and interest, and reduced sense of wellbeing and motivation have been cited. Rarefaction and thinning of pubic hair, loss of muscle and bone mass, in addition to persistent and unexplained fatigue have been mentioned as well. Most of these manifestations can be easily confused with signs of aging or overload with the rhythm of life. A biochemical characterization for the diagnosis of androgen deficiency is also not recognized. Testosterone measurement is often performed using low accuracy techniques for female serum levels. Although some laboratories already use more recent technology and better sensitivity that allow new questions about normal or reduced levels, the interpretation of such results is still controversial. Added to this is the fact that clinical effects of androgens are tissue-specific and not necessarily related to serum levels. Thus, important world societies are against the use of androgenic serum dosages as indicators of disorders resulting from the insufficiency of such hormones, especially as predictors of sexual dysfunction. 1 2 3

To reinforce this fact, studies with women undergoing bilateral oophorectomy, hence with a drop in androgen production, did not demonstrate a strong correlation between postoperative testosterone levels and sexual function. Given the difficulty in characterizing their deficiency, when would there be an indication to prescribe androgenic supplementation?

Most studies evaluating the use of testosterone have been conducted in women with sexual complaints. Evidence that female sexual function is associated with androgenic action is mainly based on studies that observed improvement in hypoactive sexual desire disorder (HSDD) in postmenopausal women treated with testosterone with or without concomitant use of conventional estrogen therapy. 4

The fact that female sexual dysfunctions are influenced by numerous clinical, surgical, interrelational and social conditions must be taken into account. Hormonal changes represent an extra factor and should be included in this context. Although the literature provides evidence of improvement in HSDD with the use of testosterone in postmenopausal women, androgenic serum levels have not been indicated as predictors of a positive or a negative outcome. According to literature data, postmenopausal women who received testosterone improved their sexual function score, although with only a slight increase in the number of satisfying sexual episodes. Likewise, testosterone replacement has shown to be effective in women undergoing bilateral oophorectomy. Thus, women with HSDD, after excluding other causes, represent the most accepted indication for testosterone supplementation. 3 4 5 6

Other debatable indications for using testosterone are for promoting wellbeing, mood and cognition. In postmenopausal women, evidence pointing to improvement in all these indications is insufficient. In this same group of women, studies also do not support the use of testosterone in physiological doses aimed at beneficial effects on bone mass or the distribution of body fat, muscle strength or body composition. 3

The benefits of androgen replacement in women during the reproductive period are also inconclusive, with divergent acceptance among world societies in the case of well-characterized HSDD. 4 7

Although interest in the use of androgens is on the rise, we emphasize that robust evidence guiding their indication is not growing at the same speed. Until new evidence emerges, we recommend that physicians choose according to each individual and prescribe androgens sparingly always using physiological doses, respecting current scientific knowledge, and guide and discuss with their patients about the relative ignorance of the risks and benefits of using androgens, in particular regarding long-term safety.

Many world societies have manifested in this direction. The National Commission Specialized in Endocrine Gynecology (Portuguese acronym: CNEGE) of FEBRASGO, the Brazilian Federation of Gynecology and Obstetrics Associations, performed an extensive literature review and pointed out their positions on this very debatable subject. These positions are being published in two articles divided according to recommendations for women's reproductive phase or climacteric with the aim to help health professionals in decision-making, whether for patient assessment or the indication of androgen therapy, its dosage and reassessment of results. 8 9
